# ARL9 is upregulated and serves as a biomarker for a poor prognosis in colon adenocarcinoma

**DOI:** 10.1186/s12876-023-02677-8

**Published:** 2023-02-23

**Authors:** Hai-deng Yang, Xiu-xiu Jin, Bin-bin Gu, Yu-Zhang, Dan Li, Ling-ling Yan

**Affiliations:** 1 Key Laboratory of Minimally Invasive Techniques and Rapid Rehabilitation of Digestive System Tumor of Zhejiang Province, Linhai, China; 2 Department of Gastroenterology, Taizhou Hospital of Zhejiang Province Affiliated to Wenzhou Medical University, Linhai, China; 3 Endoscopy Center, Taizhou Hospital of Zhejiang Province Affiliated to Wenzhou Medical University, Linhai, China

**Keywords:** ARL9, The Cancer Genome Atla, Colon adenocarcinoma, Biomarker, Prognosis

## Abstract

**Background:**

ARL9 is a newly identified member of the ARF family, and the clinical significance of ARL9 in colon adenocarcinoma is unknown. In this study, we aimed to explore the expression of ARL9 mRNA in colon adenocarcinoma, and its effect on the prognosis of patients with colon adenocarcinoma.

**Methods:**

We investigated the differential expression of ARL9 between colon adenocarcinoma tissue and adjacent tissues through a bioinformatics analysis using The Cancer Genome Atlas (TCGA) database. The correlation between clinical characteristics and the mRNA expression level of ARL9 were analyzed. A survival analysis and a Cox regression analysis were used to determine the prognostic significance of ARL9. Finally, we conducted a gene set enrichment analysis (GSEA) to explore the ARL9 signaling pathways involved in the development of colon adenocarcinoma. The effect of the expression of ARL9 on the proliferation and migration of colon adenocarcinoma was analyzed by the CCK8 method and a cell scratch test, respectively.

**Results:**

The mRNA expression of ARL9 in colon adenocarcinoma tissues was higher in comparison to the level in normal adjacent tissues (*P* < 0.05). The mRNA expression of ARL9 was not related to sex, tumor stage, T stage, N stage, M stage, but to age. The 5-year survival rate of colon adenocarcinoma patients with high ARL9 mRNA expression levels was significantly lower than that of patients with low ARL9 mRNA expression levels (*P* < 0.05). Age and the high mRNA expression of ARL9 were independent risk factors for a poor prognosis in patients with colon adenocarcinoma. The GSEA suggested that ARL9 may be able to upregulate cell adhesion, extracellular matrix receptor interactions, tumor-associated pathways, and downregulate the citrate cycle and tricarboxylic acid cycle pathway, which are involved in the development of colon adenocarcinoma. After knocking down ARL9, the proliferation and migration abilities of colon adenocarcinoma cells were decreased (*P* < 0.01).

**Conclusion:**

The mRNA expression of ARL9 is upregulated in colon adenocarcinoma, and higher mRNA expression levels are associated with a poor prognosis. Knocking down ARL9 can reduce the proliferation and migration of colon adenocarcinoma cells. ARL9 mRNA can be used as a prognostic biomarker in patients with colon adenocarcinoma.

**Supplementary Information:**

The online version contains supplementary material available at 10.1186/s12876-023-02677-8.

## Background

Colorectal cancer is the third most commonly diagnosed cancer and the second leading cause of cancer mortality. Worldwide, in 2020, more than 1.9 million new cases of colorectal cancer and 935,000 deaths were estimated to have occurred, representing approximately one in ten cancer cases and deaths [1, 2]. Among them, colorectal adenocarcinoma is the main histological subtype of colorectal cancer. The incidence of colorectal cancer is rapidly increasing due to changing lifestyles and a Westernized diet [3, 4]. In addition, the incidence of colorectal cancer increases with age, and is higher in men than in women, with 60% of colorectal cancer cases diagnosed at > 70 years of age [5]. With the development of biological knowledge, in addition to surgical treatment and radiotherapy, the development and application of targeted drugs have provided new therapeutic perspectives with chemo-free treatment strategies. The screening of key molecules through tumor tissue genetic testing, construction of prognostic prediction models, risk stratification, and finding suitable targets for targeted therapy to achieve individualized and precise treatment are crucial to improving the cure rate and reducing the tumor-related mortality.

Adenosine disphosphate (ADP)-ribosylation factor (ARF) is a member of the Ras superfamily of small GTP-binding proteins [6] and is involved in various physiological processes (e.g., cell proliferation, differentiation, migration by regulating membrane transport, and binding to the cytoskeleton) [7, 8]. Tumor cells are able to disrupt membrane transport regulators, thereby enhancing migration and invasion. Like other members of the RAS superfamily, ARF family proteins can be activated by guanine-nucleotide exchange factors (GEFs) and inactivated by GTPase-activating proteins (GAPs) [9]. When ARF family proteins are activated, they bind to effector molecules, which in turn mediate downstream functions. Members of the ARF family, including ARF-like (ARL) proteins, are associated with the development and progression of many types of cancer, including hepatocellular carcinoma [10, 11], renal cell carcinoma [12], colorectal cancer [13–17], lung cancer [14, 18], and breast cancer [13]. ARL9 is a novel GTP-binding protein that is highly conserved and widely expressed in eukaryotic cells. However, as a newly identified member of the ARF family, the clinical and prognostic significance of ARL9 in colon adenocarcinoma is unknown, and its function in colon adenocarcinoma has never been reported.

In the present study, we analyzed the differences in the mRNA expression of ARL9 in colon adenocarcinoma tissues and adjacent normal tissues in The Cancer Genome Atlas (TCGA) database and the correlation between the mRNA expression of ARL9 and patients' clinical information. The prognostic significance of ARL9 mRNA was also assessed using patient follow-up information. Finally, ARL9 was screened by a Gene Set Enrichment Analysis (GSEA) to reveal its participation in the main KEGG pathway. The aim was to investigate the functional mechanism of ARL9 in colon adenocarcinoma and provide a basis for the diagnosis, treatment and prognosis of colon adenocarcinoma.

## Materials and methods

### Data mining from public databases

We downloaded high-throughput gene sequencing data (Workflow Type: HTSeq—FPKM, Data Category: transcriptome profile) and clinical data (project ID: TCGA-COAD, Data Category: clinical) on colon adenocarcinoma from the TCGA database (https://portal.gdc.cancer.gov/). Colon adenocarcinoma tissues from a total of 398 cases and adjacent normal tissues from 39 cases were finally retrieved for the mRNA expression data, along with the clinical data of 385 colon adenocarcinoma patients, which were included in this analysis.

### The differential expression analysis

The mRNA expression levels of ARL9 in 437 colon adenocarcinoma samples and their adjacent normal tissues were compared. Patient information was then paired to compare the difference in the mRNA expression of ARL9 between tumor tissue and adjacent normal tissues in paired patients.

### Clinical association analysis

Clinical data were extracted, and after matching with sequencing data, clinical information such as age, sex, tumor stage and TNM stage were extracted and analyzed in correlation with the mRNA expression of ARL9. Then, the differences of the mRNA expression of ARL9 in tumor patients with different clinical characteristics were compared.

### Survival analysis

The colon adenocarcinoma patients were divided into high and low expression groups by the median ARL9 mRNA expression values. Expression levels above and below the median value were classified as high and low expression levels, respectively. Then, the survival of colon adenocarcinoma patients was compared between the two groups. Finally, univariate and multivariate Cox regression analyses were performed to identify independent prognostic factors for colon adenocarcinoma.

### Gene set enrichment analysis (GSEA)

Colon adenocarcinoma patients were divided into high and low expression groups based on the median ARL9 mRNA expression levels, and then an enrichment analysis of the KEGG pathway was performed using GSEA to determine the pathways through which ARL9 is involved in the development of colon adenocarcinoma [19–21].

### ARL9 knockdown experiments

#### Materials

Human colon cancer cell line HCT116 cells (Cell Resource Center, Shanghai Institutes for Biological Sciences, Chinese Academy of Sciences, ZQ0125), DMEM high-sugar medium (HyClone, SH30022.01B), opti-MEM reduced serum medium (Senrui BIO, CR-22600), lipofectamine 3000 (invitrogen, L3000001), fetal bovine serum (Bio-Channel, BC-SE-FBS06), 0.25% pancreatin (Bio-Channel, BC-CE-006), 100 × penicillin–streptomycin solution (Senrui BIO, CR-15140), 1 × PBS buffer (Senrui BIO, CR-10010), CCK8 kit (APExBIO, K1018), 6-well plates (Corning, 3516), 96-well plates (Corning, 3599), NC-siRNA (Reebok Bio, siN0000001-1-10), ARL9-siRNA-1(Reebok Bio, siG000132946A-1-5), ARL9-siRNA-2 (Reebok Bio, siG000132946B-1-5), 5 × protein loading buffer (Fude Bio, FD006), SDS-PAGE gel preparation kit (Biyuntian, P0690), 0.22-μm-pore-size pvdf membrane (Millipore, E1079), ARL9 mouse monoclonal antibody (Santa, SC-393264), Tubulin antibody (Proteintech, 11224-1-AP), HPR-labeled goat anti-mouse secondary antibody (Dawen BIO, BHR102), hypersensitivity ECL chemiluminescence detection kit (Fude BIO, FD8030).

#### ARL9 knockdown and the detection of knockdown efficiency

HCT116 cells were cultured in DMEM medium with 10% fetal bovine serum. HCT116 cells in the logarithmic growth phase were seeded in 6-well plates. After the cells had grown to 30–50%, 250 μl of opti-MEM + 6 μl NC-siRNA or (3 μl ARL9-siRNA-1 + 3 μl ARL9-siRNA-2) + 5 μl lipo3000 was added to each well for transfection. The fluid was changed 24 h after transfection. The cells were lysed with 200 μl of 1 × protein loading buffer for each well and then denatured at 95 °C for 10 min. ARL9 knockdown was confirmed by Western blotting. Proteins were separated by SDS/PAGE and transferred to polyvinylidene difluoride membranes. After being incubated in 5% nonfat milk, the membrane was incubated with primary antibodies at 4 °C overnight. Then, the membrane was incubated with secondary antibodies at room temperature for 1 h. Proteins were detected with a hypersensitivity ECL chemiluminescence detection kit. Tubulin was used as the control.

#### Detection of the colon adenocarcinoma cell proliferation capacity

HCT116 cells transfected with ARL9-control or ARL-siRNA for 24 h were centrifuged and resuspended by pancreatin digestion, and the cell suspension was diluted to a concentration of 10^4^ cells/ml after counting in a cell counting plate. Cells were then seeded in 96-well plates with 100 μl per well, and an equal amount of PBS solution in a circle of blank wells around the cell wells. A total of 4 plates were spread. The original medium was removed and replaced with culture medium containing 10% CCK8 at 1, 2, 3 and 4 days after cell seeding, and the optical density (OD) value at 450 nm was measured after continued incubation for another 4 h.

#### Detection of the colon adenocarcinoma cell migration capability

HCT116 cells transfected with ARL9-control or ARL-siRNA for 24 h were centrifuged and resuspended by pancreatin digestion, and the cells were seeded in 6-well plates at 80% confluence. When the cells grew to 100% confluence, gun head was used to perform a scratch. After adding PBS to wash away the floating cells, DMEM medium containing 5% fetal bovine serum was added, labeled and photographed under a microscope, and the culture was continued for 24 h and photographed at the same position.

### Statistical analysis

Statistical analyses were performed using the R language (version 4.0.3), which required the installation and loading of relevant packages (limma, beeswarm, ggplot2, survival, survminer, etc.). GSEA software version 3.0 was used to perform the gene set enrichment analyses. The results were expressed as bee swarm plots, box plots, survival curves, forest plots, and multi-GSEA enrichment plots. The CCK8 assay was used to measure the proliferative capacity of cells, and a two-way ANOVA was performed to compare the differences in OD values between groups at different time points. The cell scratch assay was performed to test cell migration ability, and the migration distance and healing rate were analyzed by an unpaired *t*-test. *P* values of < 0.05 were considered to indicate statistical significance.

## Results

### ARL9 is expressed at high levels in colon adenocarcinoma tissues

**Fig. 1:**
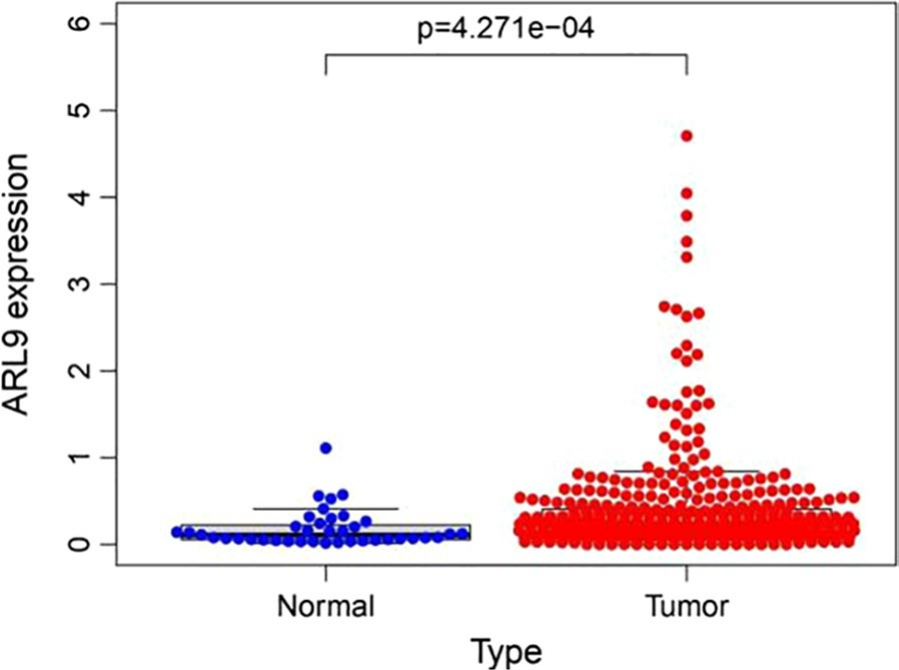
Comparison of ARL9 mRNA expression between colon adenocarcinoma tissues and adjacent normal mucosa

**Fig. 2:**
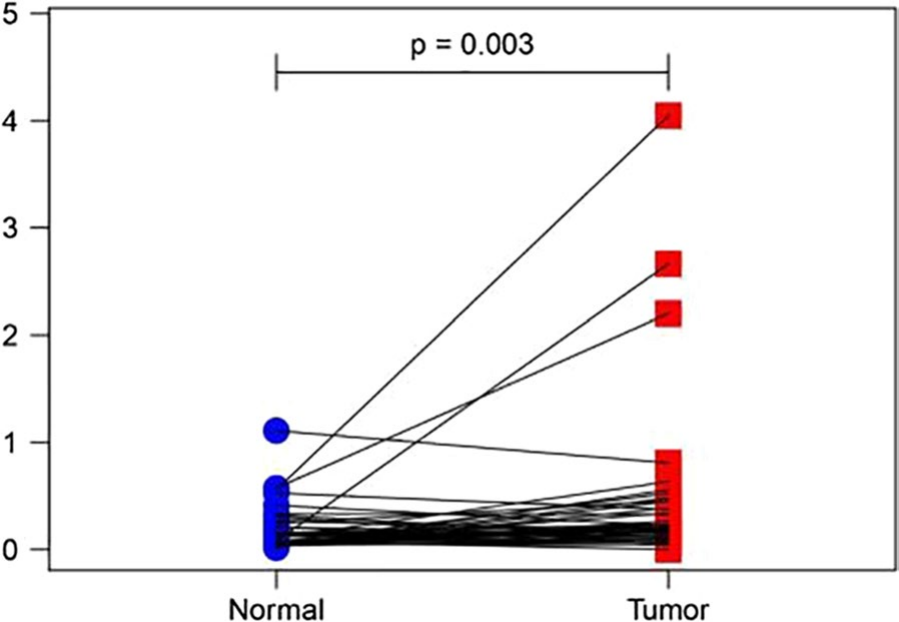
Comparison of ARL9 mRNA expression between paired colon adenocarcinoma tissues and adjacent normal mucosa

Comparison of the mRNA expression of ARL9 between colon adenocarcinoma tissues and adjacent normal mucosa in the TCGA database is shown in Figs. 1 and 2. The expression levels of ARL9 mRNA in colon adenocarcinoma tissues were higher than those in adjacent normal tissues, and the differences were statistically significant (*P* < 0.05).

### Comparison of the mRNA expression of ARL9 in colon adenocarcinoma tissues of patients with different clinical characteristics in the TCGA database

**Fig. 3:**
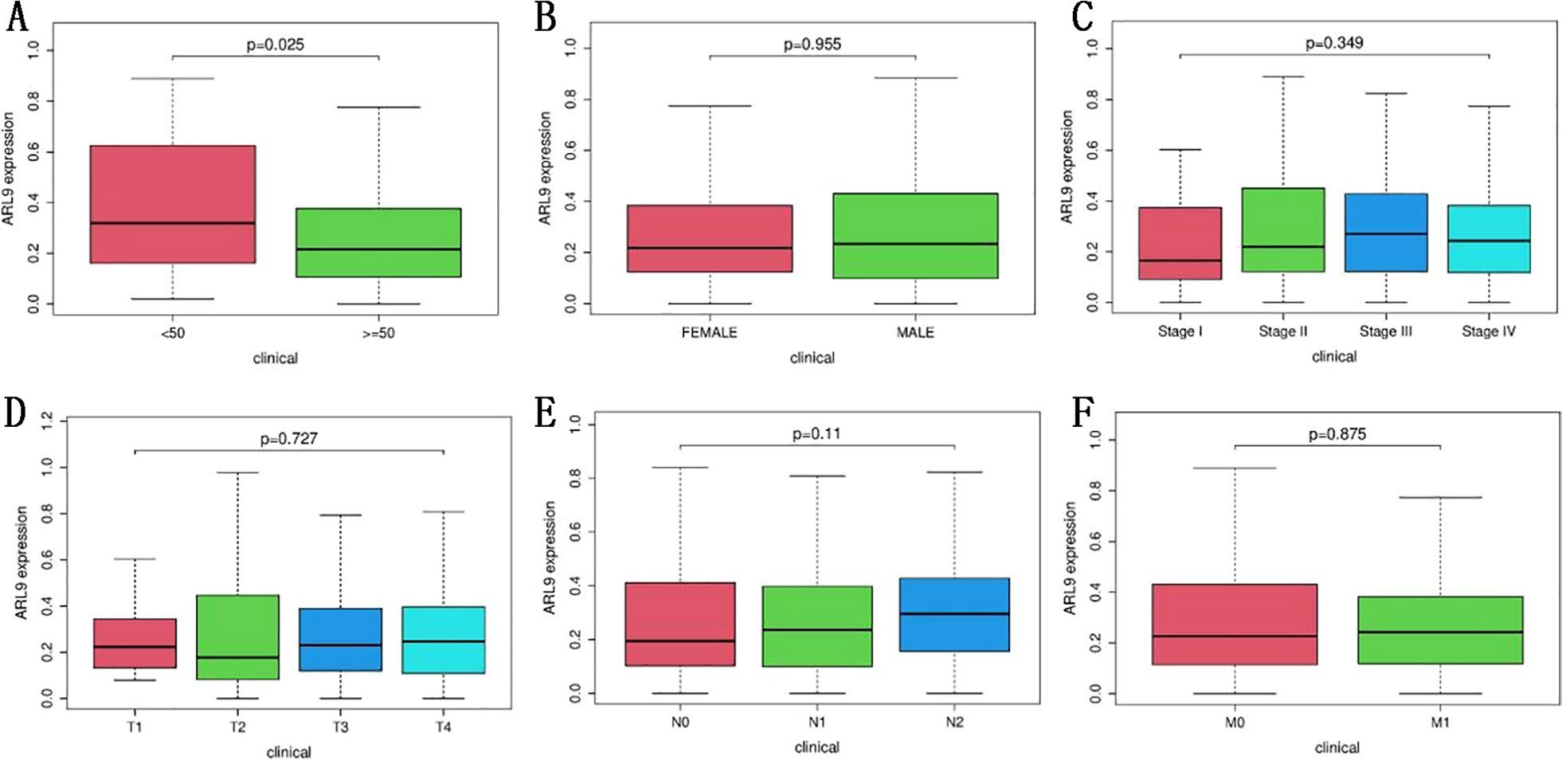
The association analysis between ARL9 mRNA expression and colon adenocarcinoma patient clinical characteristics. **A** Age **B** Sex **C** Stage **D** T stage **E** N stage **F** M stage

As shown in Fig. 3, the mRNA expression of ARL9 was lower in colon adenocarcinoma patients of ≥ 50 years of age in comparison to those aged of < 50 years of age (*P* < 0.05). Furthermore, the difference in the mRNA expression of ARL9 in colon adenocarcinoma tissues did not differ to a statistically significant extent according to sex, tumor stage, T stage, N stage, or M stage (all *P* > 0.05) (Additional file 2).

### The high expression of ARL9 is associated with a poor prognosis in patients with colon adenocarcinoma

**Fig. 4:**
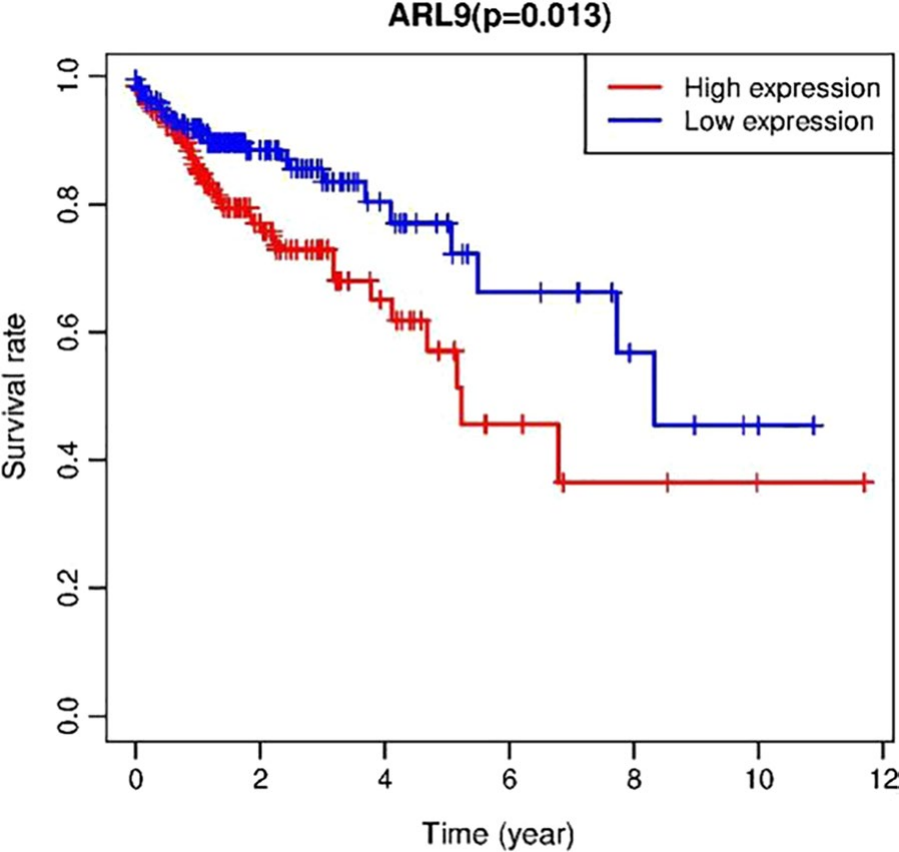
Survival curves for patients with high and low ARL9 mRNA expression in colon adenocarcinoma tissues

**Fig. 5:**
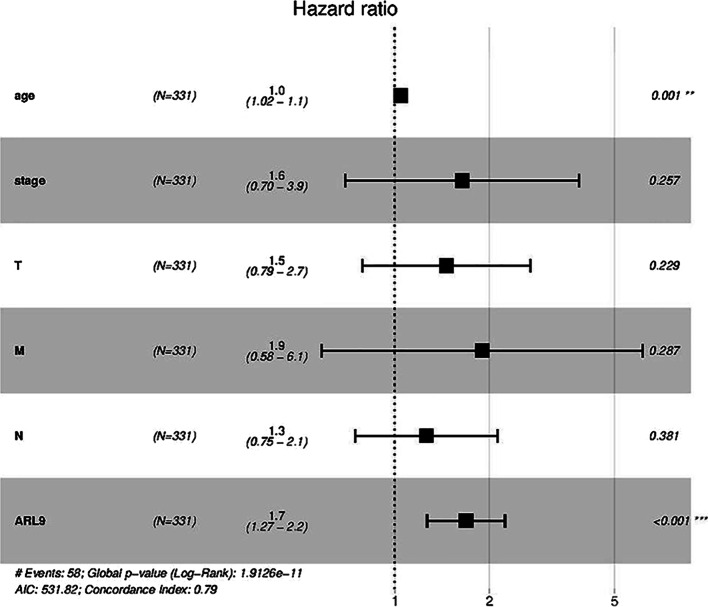
Multifactorial COX regression forest plot in patients with colon adenocarcinoma

The survival between colon adenocarcinoma patients with low and high mRNA expression levels of ARL9 from the TCGA database was compared in Fig. 4. Subsequently, age, sex, tumor stage, T stage, N stage, M stage and mRNA expression of ARL9 were included in the univariate Cox regression analysis, and variables with *P* < 0.1 were included in a multivariate Cox regression analysis, as shown in Table 1. Forest plots are shown in Fig. 5.

**Table 1:** COX regression analysis of risk factors associated with prognosis in patients with colon adenocarcinoma

Variable	Univariate analysis			multivariate analysis		
	HR	95% CI	*P* value	HR	95% CI	*P* value
Age, years	1.0299	1.0051–1.0553	0.0179	1.0430	1.0171–1.0695	0.0010
Sex	1.1265	0.6681–1.8993	0.6549			
Stage	2.5019	1.8508–3.3820	0.0000	1.6395	0.6973–3.8548	0.2571
T stage	2.9267	1.7421–4.9168	0.0000	1.4600	0.7884–2.7036	0.2287
N stage	2.1753	1.6089–2.9410	0.0000	1.2611	0.7502–2.1198	0.3814
M stage	5.2259	3.0648–8.9111	0.0000	1.8926	0.5846–6.1277	0.2872
ARL9	1.4412	1.1003–1.8877	0.0079	1.6846	1.2666–2.2405	0.0003

*HR* hazard ratio, *CI* confidence interval

As seen in Fig. 4, the 5-year survival rate of patients with high ARL9 mRNA expression levels in colon adenocarcinoma tissues was significantly lower than that of patients with low expression levels (*P* < 0.05); their 5-year survival rates were 51.4% and 72.2%, respectively.

As seen in Table 1 and Fig. 5, patient age and the high expression of ARL9 were independent risk factors for a poor prognosis, with a 1.0430-fold increase in risk per 1-year increase in age (*P* = 0.001) and a 1.6846-fold increase in risk per 1 increase in the mRNA expression of ARL9 (*P* < 0.001).

### The GSEA of tumor tissues from colon adenocarcinoma patients

**Fig. 6:**
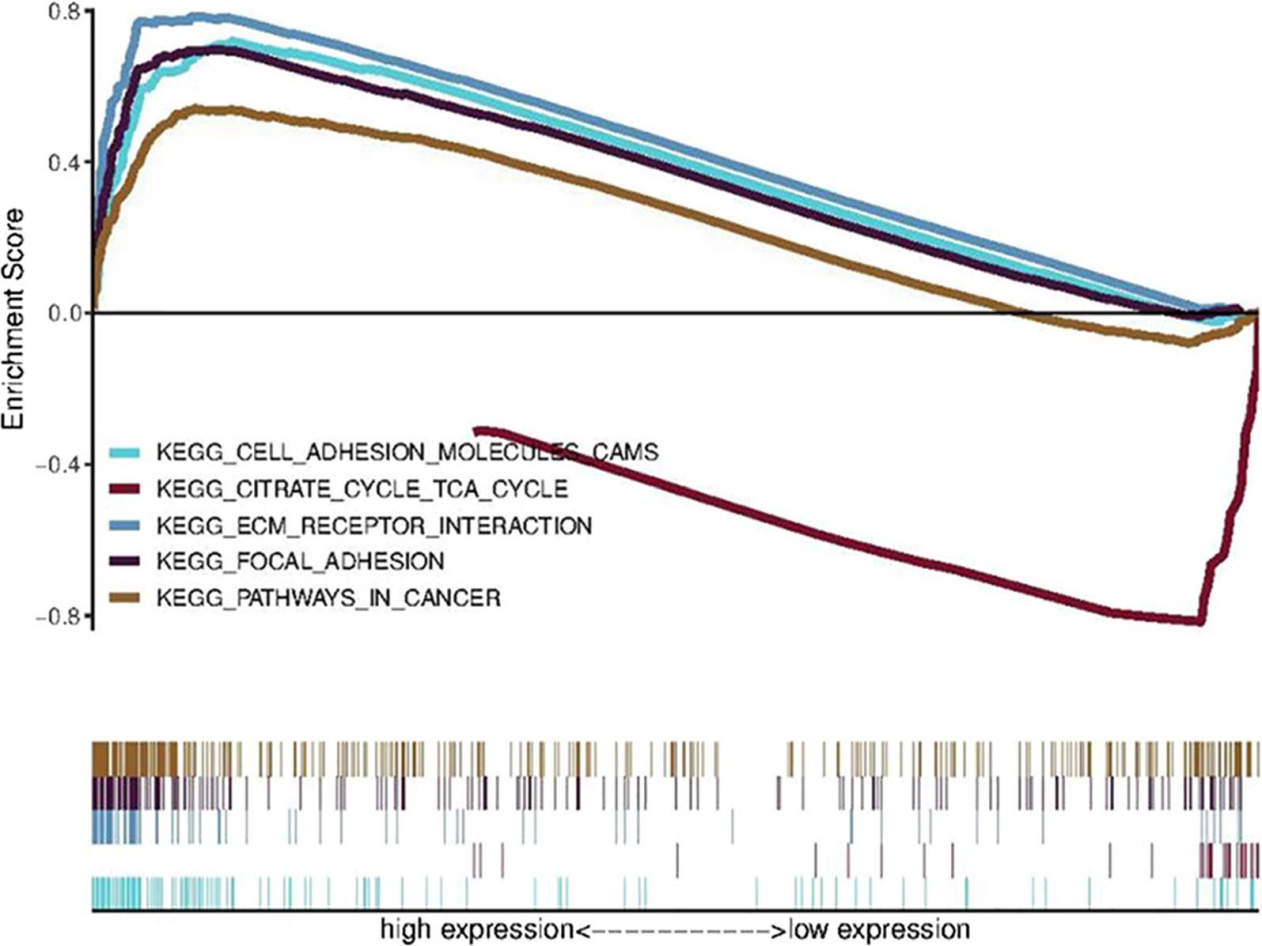
GSEA enrichment map of high and low ARL9 mRNA expression in KEGG pathway

The mRNA expression of ARL9 in colon adenocarcinoma patients was divided into low and high expression groups for the GSEA, and the target pathways were selected from the KEGG enrichment results with a false discovery rate (FDR) q value of < 0.05 in the high and low expression groups, respectively, as shown in Table 2; the multi-GSEA is plotted in Fig. 6.

**Table 2:** Gene set enrichment analysis of high and low ARL9 mRNA expression in KEGG pathway

KEGG pathway	ES	NES	NOM *p* value	FDR *p* value
Focal adhesion	0.6974	2.3296	0.0000	0.0017
Cell adhesion molecules cams	0.7193	2.2089	0.0019	0.0039
ECM receptor interaction	0.7851	2.2001	0.0000	0.0035
Pathways in cancer	0.5409	2.1440	0.0000	0.0025
Citrate cycle TCA cycle	-0.8458	-2.0625	0.0000	0.0339

*ES* enrichment score, *NES* normolized enrichment score, *NOM* nominal, *FDR* false discovery rate

As seen in Table 2 and Fig. 6, in the ARL9 mRNA high expression group, the cell adhesion (FOCAL ADHESION, CELL ADHESION MOLECULES CAMS), extracellular matrix receptor interaction (ECM RECEPTOR INTERACTION), and tumor-associated pathways (PATHWAYS IN CANCER) were enriched, indicating that the high expression of this gene may promote the development of colon adenocarcinoma through the upregulation of these pathways. In contrast, the citrate cycle and tricarboxylic acid cycle pathways were enriched in the low expression group, suggesting that this gene may inhibit this metabolic pathway in the development of colon adenocarcinoma.

### ARL9 knockdown reduced the proliferation and migration capacity

**Fig. 7:**
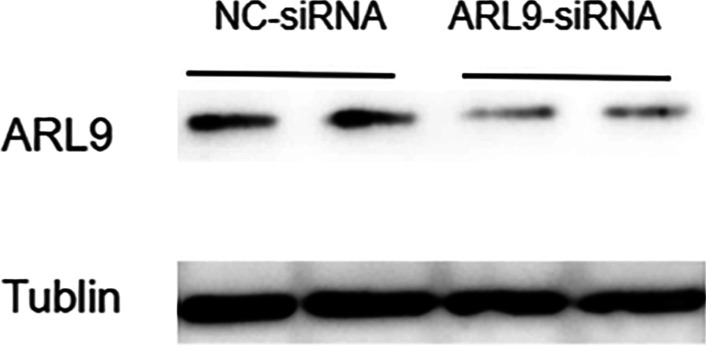
Knockdown of ARL9 in HCT116 cells

**Fig. 8:**
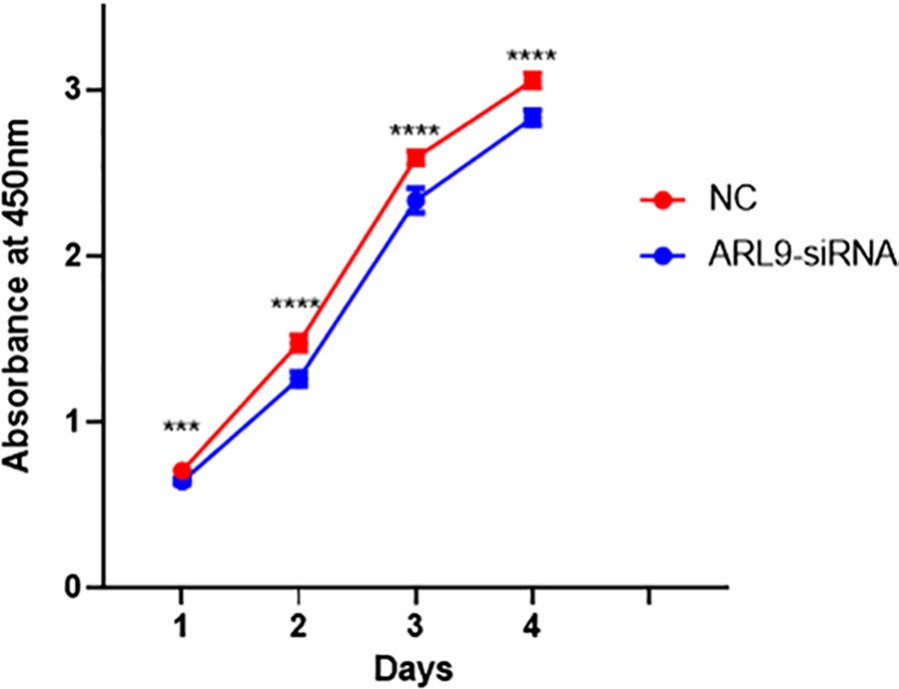
Growth curves of transfected cells

**Fig. 9:**
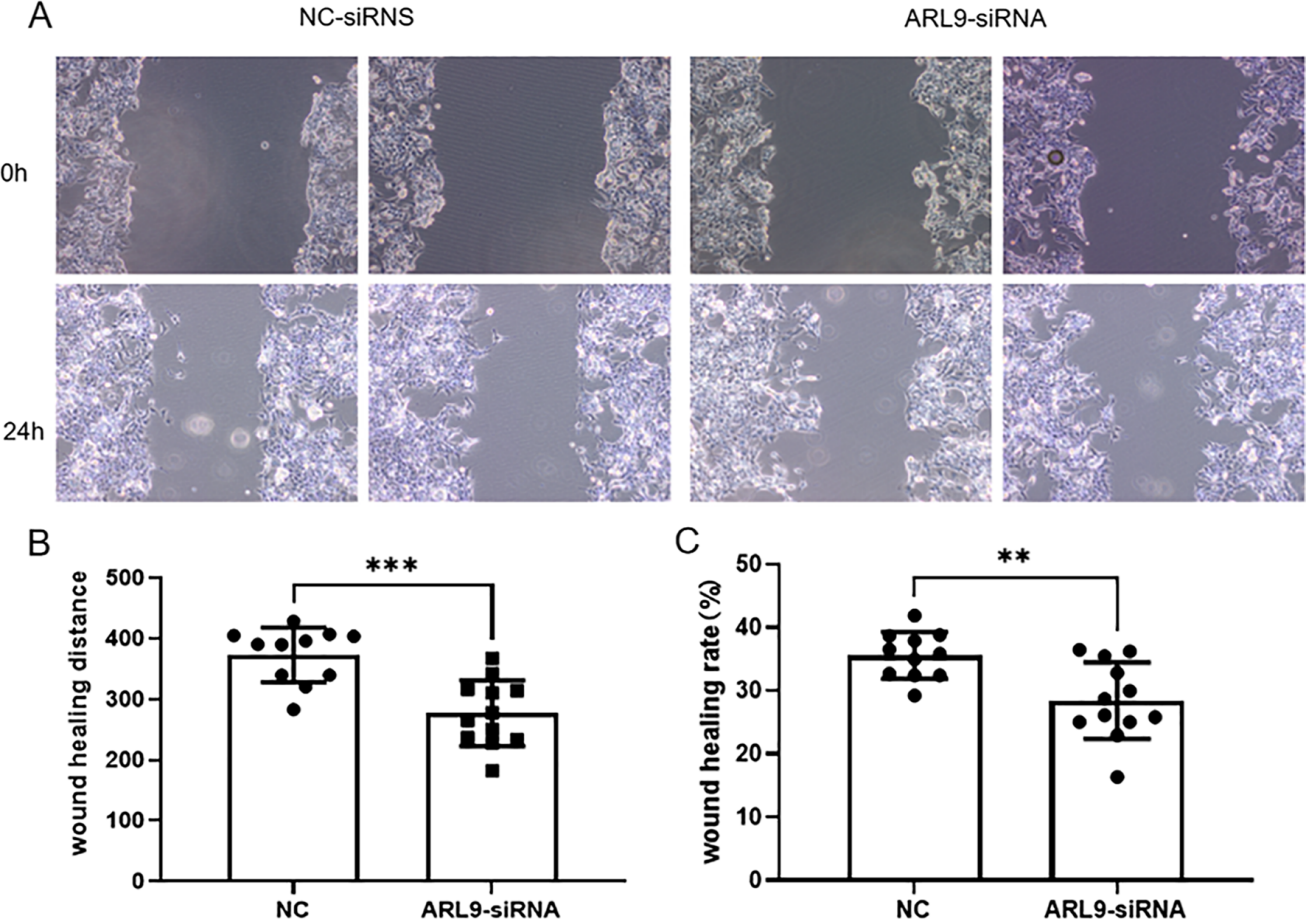
Cell migration following varied transfection measured by Scratch test. **A** Width at 0 h and 24 h after scratching. **B** Comparison of migration distance of transfected cells between two groups (0 h scratch width—24 h scratch width). **C** Scratch healing rate of transfected cells between the two groups [(0 h scratch area—24 h scratch area)/0 h scratch area]

In this study, two knockdown sequences, si-ARL9-1 and si-ARL9-2, were applied, and the latter had better knockdown efficiency, so si-ARL9-2 was applied in the subsequent experiments (Additional file 1). HCT116 cells transfected with ARL9-siRNA showed significantly lower ARL9 protein expression levels in comparison to the NC-siRNA group (Fig. 7). The CCK8 assay confirmed that the knockdown of ARL9 reduced the proliferation capacity of colon adenocarcinoma cells (Fig. 8). ARL9 knockdown attenuated the capacity of HCT116 cells for migration (Fig. 9).

## Discussion

Colon adenocarcinoma is one of the most common malignant tumors of the digestive tract worldwide. It has no obvious symptoms in the early stage and is often diagnosed in an advanced stage, which is associated with high mortality and a poor prognosis[22]. Therefore, the search for biomarkers of colon adenocarcinoma and the accurate and effective risk stratification of patients with advanced disease are important for the early diagnosis of patients and personalized and targeted treatment. The present study explored the associations between ARL9 mRNA levels and the initiation, development and prognosis of colon adenocarcinoma.

The present study demonstrated that the mRNA expression levels of ARL9 were significantly upregulated in colon adenocarcinoma tissues in comparison to adjacent normal tissues. Meanwhile, we also compared the correlation between different tumor stages, T stage, N stage, M stage and ARL9 mRNA levels. Although the difference between the group comparisons was not statistically significant, the N stage tended to be elevated, and whether it is involved in tumor progression needs to be further verified. Additionally, the present study showed that the 5-year survival rate of patients with the high mRNA expression of ARL9 was significantly lower than that of patients with low expression levels, and the high mRNA expression of ARL9 was an independent risk factor for a poor prognosis in patients with colon adenocarcinoma, as verified by the Cox regression analysis. In this study, we also performed a GSEA of ARL9 and concluded that ARL9 is highly expressed in colon adenocarcinoma tissues and that it may be involved in the development of colon adenocarcinoma through the upregulation of cell adhesion, extracellular matrix receptor interaction, and the activation of tumor-associated pathways, and the downregulation of the citrate cycle and tricarboxylic acid cycle pathways. Finally, using in vitro experiments, the present study confirmed that ARL9 knockdown in HCT116 cells (a colon adenocarcinoma cell line) reduced the cell proliferation and migration abilities.

Members of the RAS superfamily of small GTPases are the primary regulators of the various steps involved in membrane traffic. Several studies have demonstrated that disruption of membrane transport regulatory molecules in tumor cells can promote migration and invasion[9]. Previous studies have shown that the expression and/or activity of ARF family proteins and their GEFs, GAPs, and effectors are frequently upregulated in several types of cancer and play a role in cancer cell migration, invasion, and proliferation[10, 13, 14]. In particular, ARF1, ARF4, ARF6 and ARL4 are aberrantly expressed in different types of cancer cells and human cancers, (e.g., breast, gastric, prostate and lung cancer), and their expression is associated with higher grade tumors, suggesting that these ARF family proteins play a crucial role in cancer development. Therefore, we suggest that the upregulation of ARL9 mRNA may be related to the development and prognosis of colon adenocarcinoma and may promote tumor progression through membrane transport-related links, while its correlation with colon adenocarcinoma metastasis requires further study.

## Conclusion

In conclusion, ARL9 plays an important role in the occurrence and progression of colonic adenocarcinoma. The expression of ARL9 is upregulated in colon adenocarcinoma tissues, and patients with high ARL9 expression levels have a poorer prognosis. The knockdown of ARL9 reduces the proliferation and migration ability of colon adenocarcinoma cells. ARL9 can act as an independent prognostic predictor in colonic adenocarcinoma.

## Supplementary Information

**Additional file 1**. Original image of western blot.

**Additional file 2**. Clinicopathological features of patients with colon adenocarcinoma.

## Data Availability

The datasets generated and/or analysed during the current study can be accessed and obtained at the following link, https://data.mendeley.com/datasets/yhc7n9tc9w/2.

## Abbreviations

TCGA: The Cancer Genome Atlas
GSEA: Gene set enrichment analysis
ADP: Adenosine disphosphate
ADP: Adenosine disphosphate-ribosylation factor
GEFs: Guanine-nucleotide exchange factors
GAPs: GTPase-activating proteins
ARL: ARF-like
FDR: False discovery rate
