# Supplementary figures and images for: ARL9 is upregulated and serves as a biomarker for a poor prognosis in colon adenocarcinoma
Source: BMC Gastroenterol. 2023 Feb 23;23:48. doi: 10.1186/s12876-023-02677-8 (PMC9951453; doi:10.1186/s12876-023-02677-8)

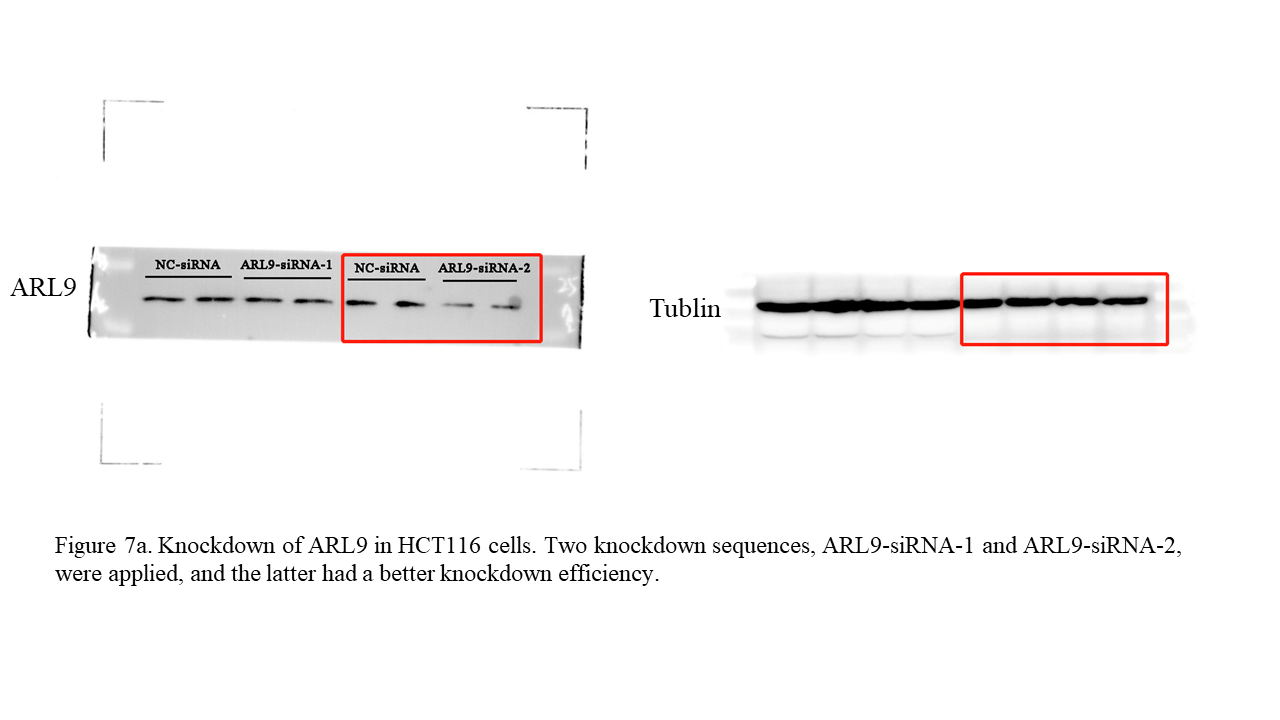

Supplement: Supplementary file 1 — Additional file 1. Original image of western blot. [file 12876_2023_2677_MOESM1_ESM.tif]
